# RhoB modifies estrogen responses in breast cancer cells by influencing expression of the estrogen receptor

**DOI:** 10.1186/bcr3377

**Published:** 2013-01-22

**Authors:** Claire Médale-Giamarchi, Isabelle Lajoie-Mazenc, Emilie Malissein, Elise Meunier, Bettina Couderc, Yann Bergé, Thomas Filleron, Laura Keller, Claudine Marty, Magali Lacroix-Triki, Florence Dalenc, Sophie F Doisneau-Sixou, Gilles Favre

**Affiliations:** 1 INSERM U563 and UMR1037, Institut Claudius Regaud, 20-24 rue du pont St Pierre, Toulouse cedex 31052, France; 2 Faculté des Sciences Pharmaceutiques, Université Paul Sabatier Toulouse III, Toulouse cedex 31062, France; 3 EA4553, Institut Claudius Regaud, 20-24 rue du pont St Pierre, Toulouse cedex 31052, France; 4 Département de Biologie et de Pathologie, Institut Claudius Regaud, 20-24 rue du pont St Pierre, Toulouse cedex 31052, France; 5 Tumorbiologischen Labor, Klinikum der Ludwig-Maximilians-Universität, Maistraße 11, München 80337, Germany

## Abstract

**Introduction:**

RhoB has been reported to exert positive and negative effects on cancer pathophysiology but an understanding of its role in breast cancer remains incomplete. Analysis of data from the Oncomine database showed a positive correlation between *RhoB *expression and positivity for both estrogen receptor alpha (ERα) and progesterone receptor (PR).

**Methods:**

This finding was validated by our analysis of a tissue microarray constructed from a cohort of 113 patients and then investigated in human cell models.

**Results:**

We found that RhoB expression in tissue was strongly correlated with ERα and PR expression and inversely correlated with tumor grade, tumor size and count of mitosis. In human breast cancer cell lines, RhoB attenuation was associated with reduced expression of both ERα and PR, whereas elevation of RhoB was found to be associated with ERα overexpression. Mechanistic investigations suggested that RhoB modulates ERα expression, controlling both its protein and mRNA levels, and that RhoB modulates PR expression by accentuating the recruitment of ERα and other major co-regulators to the promoter of *PR *gene. A major consequence of RhoB modulation was that RhoB differentially regulated the proliferation of breast cancer cell lines. Interestingly, we documented crosstalk between RhoB and ERα, with estrogen treatment leading to RhoB activation.

**Conclusion:**

Taken together, our findings offer evidence that in human breast cancer RhoB acts as a positive function to promote expression of ERα and PR in a manner correlated with cell proliferation.

## Introduction

Hormone therapy is recommended in breast cancers that express estrogen receptor alpha (ERα) and/or progesterone receptor (PR). This therapy is largely effective but there are nevertheless many cases of systemic resistance. A number of studies have addressed the question of the mechanisms of resistance to hormone therapy [1,2]. ERα transcriptional effects are not only determined by ligands as estradiol (E_2_), but also by crosstalk between ERα and growth factor signaling [3]. The hierarchy among these associations is not known and various growth factor receptors are likely to be required [3].

Prenylated proteins such as Rho GTPases are key elements in growth factor signal transduction pathways [4]. A variety of growth factors present in the tumor microenvironment activate Rho proteins [5], especially RhoB [6-8]. As a Rho protein, RhoB cycles between GTP and GDP bound states, forming interactions with a variety of effectors that modulate activity and influence important processes in cancer [9]. RhoB, in contrast to its relatives RhoA and RhoC, has been shown to function as a tumor suppressor gene on the basis of investigations of genetically RhoB-deficient strains [10] and in human cancer cells [11-13]. RhoB is an immediate early response gene that is induced by a variety of stimuli, including growth factors [6,14-21]. Although no mutation of Rho GTPases have been detected in human tumors, a correlation has been demonstrated between Rho protein overexpression and poor clinical outcome in breast cancers [22]. RhoB overexpression has been correlated to disease progression [23], although this is a controversial issue [24], and overexpression of guanine exchange factors (GEF) for Rho GTPases have been correlated to prognosis in breast cancers [25]. Indeed, breast tumor progression is accompanied by a decrease in expression of the pro-oncogenic RhoGEF Tiam1 [26]. Moreover, expression of Rho-GDI α, a negative regulator of Rho proteins, is reported to correlate with the outcome of patients with breast cancer treated by adjuvant chemotherapy [27]. From a molecular point of view, scaffold proteins involved in Rho functions, such as Rho-GDI or Dblx, have been observed as part of ERα-containing complexes [28,29] with direct interaction between ERα and Rho-GDI [30].

Besides the suggested specific involvement of RhoB in ER signaling, there have been no detailed investigations in breast cancer cells, including the assessment of any correlation with the expression of hormone receptors in tumors. A major goal of the present study was therefore to determine the involvement of RhoB in hormone-dependent breast cancers and to investigate hypothesized crosstalks between RhoB and ERα signaling.

## Materials and methods

### Immunohistochemical analysis of tissue microarrays

A tissue microarray was constructed from the 113 patients described in Table S1 in Additional file 1. As detailed in Additional file 2, the clinical trial was conducted about 30 years ago with no consent required at that time. Cores (600 μm diameter) of histologically confirmed invasive breast carcinomas were extracted from the original paraffin blocks and re-embedded into a gridded recipient paraffin block using a tissue arrayer (Alphelys; Beecher Inc., Plaisir, France). For each case, three tumor cores and one normal breast core were taken from the original block.

Tissue microarray immunostaining was performed on a Techmate Horizon™ slide processor (Dako, Trappes, France **{ }**). Sections were incubated with antibodies to ERα (NCL-L-ER-6F11; Novocastra, Nanterre, France), PR (PgR636 clone; Dako) and RhoB (sc-180; Santa Cruz Biotechnology, Heidelberg, Germany). The ERα and PR status was classified by immunohistochemistry expression as positive (≥ 10% immunoreactive cells) or negative (< 10% immunoreactive cells), according to the standards applied in France, as recommended by the Groupe d'Evaluation des Facteurs Pronostiques par Immunohistochimie dans les Cancers du Sein. RhoB immunostaining was analyzed by evaluation of the percentage of tumor-stained cells and staining intensity, allowing assessment of an ImmunoReactive Score:

IRS=% score × intensity score

Correlation of RhoB expression with clinical variables was assessed using Mann-Whitney and Spearman's rank tests. Univariate survival analysis was performed for disease-free survival by applying the log-rank test to RhoB expression levels stratified by median value. The Kaplan-Meier method was used to link the disease-free survival according to RhoB expression in the tumors.

### Cell culture and reagents

The human breast adenocarcinoma cell lines MCF-7, ZR75, T47D, SK-BR-3 and MDA-MB-231 were obtained from the ATCC (Molsheim, France). Cells were grown routinely in DMEM (Lonza Levallois-Perret, France), supplemented with 5% fetal bovine serum (FBS; Pan-Biotech, Aidenbach, Germany). Hormonoresistant LCC2 cells (R Clarke, Karmanos Cancer Center, provided the parent MCF-7) were grown in F-12 (Lonza) phenol red-free medium, containing 5% dextran-coated charcoal (DCC)-treated FBS. Cryopreservation of cell cultures ranged from passage 1 to 10. Cells were used for experiments during up to 20 passages. Mouse embryonic fibroblasts (MEFs) were grown in DMEM with 10% FBS.

Where indicated, cells were deprived of E_2 _by growing them in phenol red-free medium containing 5% DCC-treated FBS. Cells were treated with 50 nM E_2 _or 50 ng/ml epidermal growth factor or 2 mM ICI-182, 780 (Sigma-Aldrich Chimie, Lyon, France).

### siRNA transfection

Transient transfection of 40 nM siRNA was performed using Oligofectamine^® ^(Life Technologies - Invitrogen, Saint Aubin, France) according to the manufacturer's instructions. The siRNA sequences of the oligonucleotide duplexes (Eurogentec, Angers, France) were siB1 (5'-CCGUCUUCGAGAACUAUGU-dTdT-3') and siB2 (5'-UGAUAUCCCUUGUCUGUAA-dTdT-3'), siER (5'-GGGAGAAUGUUGAAACACA-dTdT-3') and the nonspecific sequence siControl (5'-GACGUGGGACUGAAGGGGU-dTdT-3').

### Adenoviral constructs expressing RhoB and transduction protocol

Replication-defective (ΔE1, E3) adenoviral vectors expressing RhoB under the transcriptional control of the CMV promoter were constructed with the Adeasy System (Qbiogen, Illkirch, France) as described previously [31]. Cells were transduced with adenoviral vectors (control empty vector or expressing RhoB) at a multiplicity of infection of 300:1.

### Western blot analysis of human cell lines

Cells were lysed and protein analyzed by SDS-PAGE [18]. Antibodies were used against RhoB (sc-180), ERα (sc-543) and, ERβ (sc-53494) from Santa Cruz Biotechnology, phospho-ERα (Ser^118^, 2511 and Ser^167^, 2514) from Cell Signaling (Saint Quentin Yvelines, France), β-actin (MAB1501) from Chemicon (Merck Millipore; Darmstadt, Germany), and secondary antibodies were anti-mouse (MP21120) and anti-rabbit (MP23145) horseradish peroxidase from Interchim (Montluçon, France) using a chemiluminescence detection kit (ECL; Pierce, Thermo Fisher Scientific, Courtaboeuf, France). Protein abundance was quantified by Image Quant TL analysis (GE Healthcare, Velizy-Villacoublay, France).

### Analysis of mouse embryonic fibroblasts generated from RhoB-deficient mice

Heterozygous (+/-) and homozygous (-/-) mutant strains of RhoB-deficient mice [10] were kindly provided by G Prendergast (Lankenau Institute for Medical Research, Wynnewood, PA, USA). Claudius Regaud Institute animal ethics committee approval was obtained (# ICR-2012-001-A) for the use of the animal model and the study protocols. Mice were housed in polycarbonate cages in controlled conditions. MEFs were generated [10] before lysis [18]. Protein were extracted and analyzed as described above with antibody against murine ERα (sc-542) or RhoB (sc-180; Santa Cruz Biotechnology).

### GST pull-down assay

The level of activated RhoB and GTP-bound RhoB protein was measured using the GST fusion protein containing the Rho binding domain of Rhotekin [8]. The amount of GTP-bound RhoB and the total amount of RhoB in cell lysates were determined by western blot as described above [32].

### Immunocytochemistry

**Figure 1:**
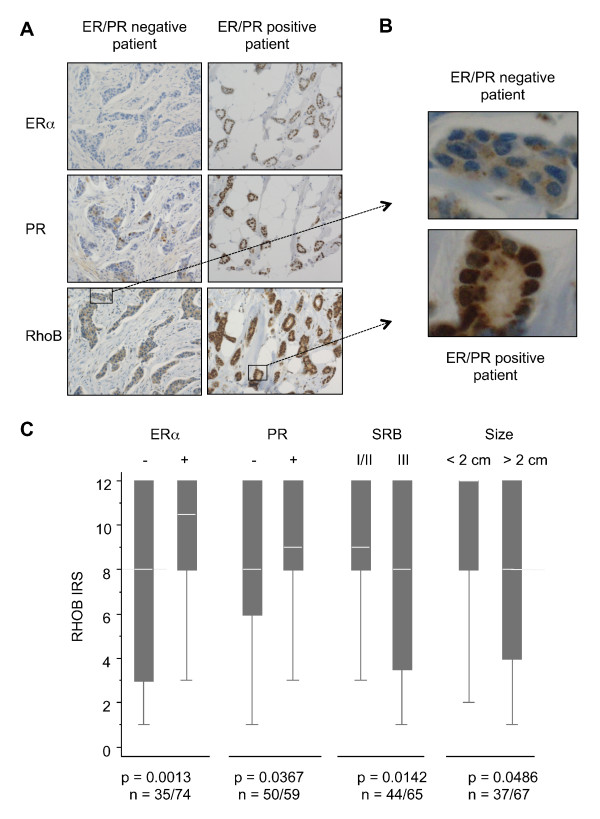
**RhoB, estrogen receptor alpha and progesterone receptor expression in tumor samples of breast cancer patients**. **(A) **Immunohistochemistry staining shown for two patients, representative of both the estrogen receptor (ER)/progesterone receptor (PR)-negative and ER/PR-positive populations (×200). **(B) **Enlargement of the squares represented in (A) (RhoB staining). **(C) **Medians of RhoB ImmunoReactive Score (IRS) scores according to ERα and PR positivity, tumor histological grade and tumor size.

For each condition, 5 × 10^5 ^cells were seeded onto glass slides dishes and were grown for 3 days in phenol red-free DMEM, containing 5% DCC-treated FBS. Cells were then treated with E_2 _during 16 hours, washed in PBS and fixed in RCL2 [33] for 2 minutes. Staining was performed by a Techmate Horizon™ slide processor as described for Figure 1. The primary antibody used was a monoclonal anti-ERα antibody (HC-20, sc-543, dilution 1:50; Santa Cruz Biotechnology) or an anti-PR antibody (PgR636 clone, dilution 1:50; Dako).

The level of ERα and PR staining was determined by evaluation of the percentage of tumor-stained cells and staining intensity, allowing assessment of an IRS.

### Quantitative reverse-transcribed PCR

Total RNA was isolated 16 hours after stimulation by E_2_, extracted using the RNeasy kit following the manufacturer's instructions (Qiagen, Courtaboeuf, France) and reverse-transcribed using the iScript™ cDNA Synthesis Kit (Biorad, Marnes la Coquette, France). Quantitative PCR was performed with an iQreal-time PCR detection system (Biorad). The specific human primer pairs used were: for GAPDH, 5'-TGCACCACCAACTGCTTAGC-3' and 5'-GGCATGGACTGTGGTCATGAG-3'; for 28s, 5'-TCGCTGGGTCTTGGATGTC-3' and 5'-AGCAGATTGTGACAGACCATTCC-3'; for PR, 5'-CGCGCTCTACCCTGCACTC-3' and 5'-TGAATCCGGCCTCAGGTAGTT-3'; and for ERα, 5'-CCACCAACCAGTGCACCATT-3' and 5'-GGT CTTTTCGTATCCCACCTTTC-3' (Eurogentec).

### Luciferase assay

Development of stable transfectants of MCF-7 cells (MELN cells) has been described previously [34]. These cells, kindly provided by P Balaguer (INSERM 540, France), were established by transfecting MCF-7 cells with ERE-β-globin-luc-SV-Neo plasmid and thus express luciferase in an estrogen-dependent manner.

Three days after siRNA transfection, MELN cells were seeded in DMEM-DCC-treated FBS during 3 days. They were then treated with E_2 _or ethanol for 16 hours. Cells were then lysed in reporter lysis buffer (Promega, Charbonnières Les Bains, France). The luciferase activity was measured with luciferase assay reagent (Promega) according to the manufacturer's instructions. Protein concentration was measured to normalize the luciferase activity data.

### Chromatin immunoprecipitation assay

Chromatin immunoprecipitation assays were performed with modifications of the procedure of Metivier and colleagues [35] as described elsewhere [36]. Briefly, 10^6 ^cells were synchronized by 3 days of culture in DMEM 5% DCC-treated FCS and treated during 1 hour with E_2_. Immunoprecipitation antibodies were ERα (sc-543), HDAC1 (sc-6299), and polII (sc-899) from Santa Cruz Biotechnology, and acetylated histone H3 (ab1791) and H4 (ab193) from Abcam (Paris, France). Quantitative PCR were performed on an iCycler (Biorad) using the primers 5'-GGCGACACAGCAGTGGGGAT-3' and 5'-TCTCCTCCCTCTGCCCCTATATTC-3' (Eurogentec) to amplify the fragment of the human PR promoter flanking the +745 AP-1 site [37].

### Cell growth determination

Forty-eight hours after siRNA transfection or transduction, cells were seeded in DMEM 5% FBS with ethanol or E_2 _and counted daily during the next 4 days with a coulter counter (Beckman Coulter, Grenoble, France).

## Results

### RhoB expression correlates positively with receptor status and negatively with grade in human breast tumors

Using the Oncomine microarray database, a correlation between *RhoB *and either ERα or PR expression was documented in datasets from 19 breast cancer studies. Moreover, four studies showed an inverse correlation between *RhoB *expression and tumor grade. To confirm the clinical relevance of RhoB expression levels in breast cancer oncogenesis and outcome, we compared the expression levels of RhoB in breast carcinomas from a cohort of 113 patients treated or not by tamoxifen in an adjuvant setting in a randomized prospective study. Patient characteristics are described in Table S1 in Additional file 1. ERα and PR assessments were initially performed with biochemistry techniques at the time of diagnosis (between 1980 and 1983). They have all been performed again at the time of analysis, in parallel with RhoB assessment. After new pathological analysis, 65 tumors were classified as grade I and II, 74 were ERα-positive, and 59 were PR-positive. Among these tumors, 23 presented a lymphovascular invasion and 39 cases presented with positive lymph nodes. After randomization, we determined that age, ERα and PR status, histological grade, and type and lymphovascular invasion status were similar in the two groups of patients, whether treated or not. Nonetheless, patients treated with tamoxifen (*n *= 62) had less favorable prognostic factors regarding positive lymph nodes, pathological tumor size and number of mitoses.

Figure 1A shows the RhoB immunohistochemistry stainings of two representative tumors of patients displaying opposite hormone receptor status (ERα/PR-negative and ERα/PR-positive), illustrating the direct correlation between RhoB and both ERα and PR expression. Enlargement of the photographs (Figure 1B) illustrates in the tumor cells of the ERα/PR-negative patient that RhoB is present and specifically cytoplasmic, although the staining is weak. For the ERα/PR-positive patient, in addition to cytoplasmic staining, a strong staining was also observed at nucleus level.

Statistical analysis of the results indicated that RhoB expression in tumors is strongly correlated with the percentage of ERα (Spearman's ρ = 0.3659, *P *= 0.001) and PR (ρ = 0.2544, *P *= 0.0076) expression, but inversely correlated with histological tumor size (ρ = -0.2344, *P *= 0.0166) and number of mitoses (ρ = -0.2009, *P *= 0.0362). We divided the patients with ERα-positive tumors into two groups with either low or high RhoB expression, and in each group we further divided the patients into groups that did or did not receive tamoxifen. This analysis argued that the level of RhoB expression was not correlated with disease-free survival of patients with ERα-positive tumors, regardless of tamoxifen treatment or not (Kaplan-Meier curve shown in Figure S1 in Additional file 3).

The RhoB IRS (see Materials and methods) integrating both the percentage and intensity of the staining (Figure 1C) was significantly higher in ERα-positive tumors (median 10.5 (3 to 12)) as compared with ERα-negative tumors (median 8 (1 to 12)), in PR-positive tumors (median 9 (3 to 12)) as compared with PR-negative tumors (median 8 (1 to 12)), and in patients with tumor grade I and II (median 9 (3 to 12)) as compared with grade III (median 8 (1 to 12)). The line in the center of each box represents the median value of the distribution, and the upper and lower ends of the box are the upper and lower quartiles, respectively. The RhoB level of expression was also higher in the smaller tumor size (≤ 2 cm, median 12 (2 to 12)) as compared with larger tumors (> 2 cm, median 8 (1 to 12)). The RhoB IRS score was not correlated with the presence of lymphovascular invasion (*P *= 0.26), nor with the presence of lymph node invasion (*P *= 0.74).

### RhoB regulates ERα expression in MCF-7, *in vivo *and in other breast cancer cell lines

**Figure 2:**
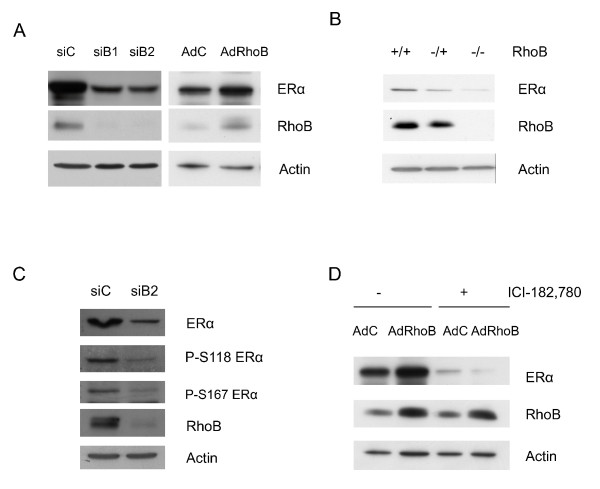
**Regulation of estrogen receptor alpha expression by RhoB in MCF-7 cells and *in vivo***. **(A) **Cells were transfected with siControl (siC), siB1 or siB2 (left panel) or transduced with adenoviral vectors (multiplicity of infection (MOI) 300:1) (right panel) during 48 hours. **(B) **Mouse embryonic fibroblasts (MEFs) of RhoB-deficient mice were lysed. **(C) **MCF-7 cells were transfected with siC or siB2. Estrogen receptor alpha (ERα) phosphorylation was analyzed 48 hours later. **(D) **Cells were transduced with adenoviral vectors (MOI 300:1) during 48 hours, and 3 days later were treated by ICI-182, 780 or ethanol during 16 hours. (A) to (D) Protein expression was analyzed. Representative of three independent experiments. AdC, adenoviral control empty vector.

We confirmed the expectation of an effect of RhoB on ERα expression in MCF-7 cells in the presence of E_2_, using two different siRNA sequences to target RhoB mRNA (siB1 and siB2) (Figure 2A, left panel). We observed associated decreases in ERα expression of 60% and 62% (siB1 and siB2, respectively). This result was confirmed using two other independent siRNA sequences targeting RhoB (data not shown).

To rule out the possibility of off-target effects of the siRNA approach, we transduced MCF-7 cells with an adenoviral vector expressing RhoB (Figure 2A, right panel). The observed 4.5-fold overexpression of RhoB increased the expression of ERα (186%), further supporting the hypothesized regulatory relationship.

We extended the study of RhoB downregulation on ERα expression to additional cell lines (Figure S2 in Additional file 4), confirming a decrease of ERα expression with RhoB depletion using siB1 and siB2 in T47D and ZR75 cells (hormone-dependent cells) or in LCC2 cells (hormone-resistant cells). We confirmed the involvement of RhoB on ERα expression *in vivo *using mice that are genetically deficient in RhoB (Figure 2B). A major decrease of ERα expression was visualized in MEFs collected from RhoB^+/- ^mice with an even more dramatic decrease in MEFs collected from RhoB^-/- ^mice. These results extended the support for a hypothesized regulatory relationship between RhoB and ERα.

We also studied the levels of ERα phosphorylated forms whose involvement as prognostic markers is discussed below (Figure 2C). We observed that the decrease of ERα expression observed when RhoB expression is downregulated is not associated with any significant specific change of P-Ser^118 ^or P-Ser^167 ^ERα level. Indeed, the ratio phosphorylated/total ERα is not modified after RhoB inhibition.

We then demonstrated that ERα expression is dramatically decreased in the presence of the pure anti-estrogen ICI-182, 780, even when RhoB is overexpressed (Figure 2D).

### Estrogen induces RhoB activation

**Figure 3:**
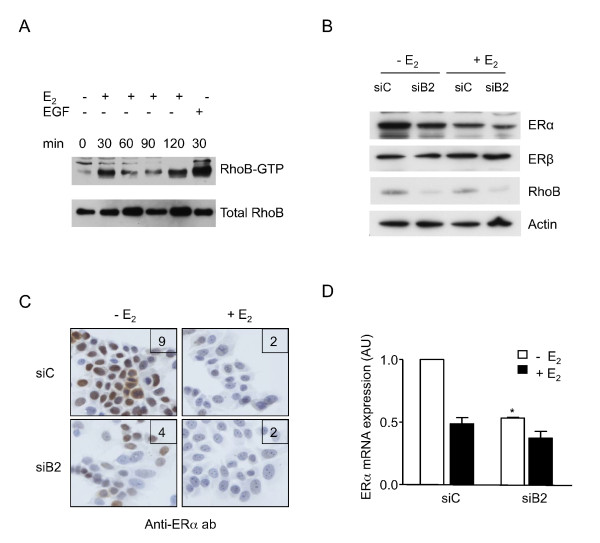
**RhoB activation and regulation of estrogen receptor alpha expression with/without estradiol in MCF-7 cells**. **(A) **Cells were deprived of estradiol (E_2_) for 3 days and then treated by E_2 _or epidermal growth factor (EGF) during the indicated times. GTP-bound RhoB and total lysates were collected. Protein expression was then analyzed. Representative of three to four independent experiments. (B) to (D) Cells were transfected with siControl (siC) or siB2, and 3 days later were deprived of E_2 _for 3 additional days and treated for 16 hours with E_2 _or ethanol. **(B) **Protein expression was then analyzed. Representative of three independent experiments. **(C) **Estrogen receptor alpha (ERα) expression was analyzed by immunocytochemistry. ImmunoReactive Score (IRS) shown in the upper-right corner. Representative of three independent experiments. **(D) **Expression of the *ERα *gene was then measured. Error bars represent the mean values ± standard deviation from triplicate conditions, representative of two independent experiments. Differences were considered statistically significant at *P < 0.01, Student's *t *test.

In examining the effect of estrogen on RhoB expression and activity and given that the activation of RhoB is important for its physiological effect [8,18,20], we investigated the role of estrogen stimulation on the RhoB guanine nucleotide binding status (Figure 3A) to analyze rapid effects of E_2 _stimulation on RhoB activation as well as expression. We observed that RhoB is quickly activated at 30 minutes without any change of total RhoB expression, as described with epidermal growth factor stimulation [8,20]. At 1 hour and 2 hours of treatment, E_2 _rapidly increased the total RhoB expression with a second peak of GTP-bound RhoB occurring at 2 hours associated with a parallel increase of total RhoB expression.

Together these results suggested that RhoB and estrogen signaling are integrated into a feed-forward loop that may positively modify the biological effects of estrogen treatment.

### RhoB regulates ERα, but not ERβ, expression in the absence and presence of estradiol in MCF-7 cells

We analyzed further the effect of RhoB downregulation on ERα and ERβ expression in the absence and presence of E_2 _(Figure 3B). As expected, E_2 _treatment induced a major significant decrease of ERα expression and concomitantly of RhoB expression. In the presence of siB2, a 36 to 41% decrease of ERα expression was observed in the absence of E_2 _and 48 to 52% in the presence of E_2_. In contrast, ERβ expression was not clearly altered by RhoB downregulation (Figure 3B). Using immunocytochemistry, we confirmed these results suggesting that RhoB regulates ERα expression and observed no dramatic changes of RhoB subcellular localization within MCF-7 cells (Figure 3C). ERα was highly concentrated in the nucleus of the untreated control with a significant staining of the related cytoplasm. In the presence of E_2_, the staining intensities of both nuclei and cytoplasms were clearly decreased with no more detectable staining in the cytoplasm, confirmed by IRS assessment. For siB2-treated cells, a similar major decrease of the labeling intensity was observed in the cytoplasm and the nucleus, both in the presence and the absence of E_2_. In the presence of E_2_, the IRS score was maintained at a value of 2 because, in spite of the difference from 30 to 20% of stained cells induced by siB2 treatment, the intensity is kept very low in both cases (value of 1).

We then investigated whether RhoB downregulation modulates ERα mRNA expression (Figure 3D). We first confirmed the expected decrease of ERα mRNA expression in control cells treated by E_2 _alone (to 0.49). In the cells treated with siB2, a dramatic decrease of ERα mRNA was observed in the absence of E_2 _(from 1 to 0.53) and much lighter in the presence of E_2 _(from 0.49 to 0.37).

### RhoB promotes ERα transcriptional activation function induced by estrogen

**Figure 4:**
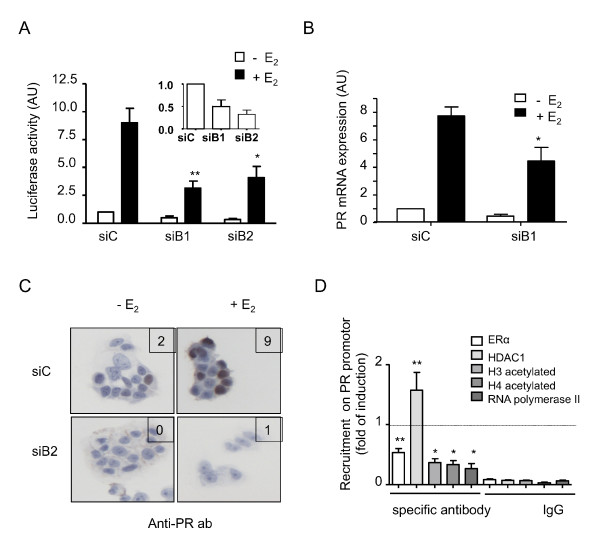
**RhoB downregulation effect on estrogen receptor alpha-dependent transcription and recruitment of cofactors on *PR *promoter**. MELN or MCF-7 cells were transfected with siControl (siC), siB1 or siB2, and 3 days later were deprived of estradiol (E_2_) for 3 additional days. **(A) **MELN cells were then treated for 16 hours with E_2 _or ethanol. Luciferase activity was quantified and normalized. Error bars represent the mean values ± standard deviation (SD) from four independent experiments. Data generated in the absence of E_2 _are enlarged in the upper-right corner. **(B) **MCF-7 cells were then treated for 16 hours with E_2 _or ethanol and expression of the *PR *gene was measured. Error bars represent the mean values ± SD from three independent experiments. **(C) **MCF-7 cells were then treated for 16 hours with E_2 _or ethanol and progesterone receptor (PR) expression was analyzed by immunocytochemistry. ImmunoReactive Score (IRS) shown in the upper-right corner. Representative, respectively, of four and three independent experiments. **(D) **MCF-7 cells were treated for 1 hour with E_2 _and lysed for chromatin immunoprecipitation (ChIp) experiments using indicated antibodies. Specificity of the immunoprecipitation was controlled using nonspecific rabbit IgG. Recruitment of these proteins on the *PR *promoter was quantified. The fold induction for the recruitment of siB1 versus siC conditions was calculated. Error bars represent the mean values ± SD from three independent experiments. Differences were considered statistically significant at *P < 0.01 and **P < 0.001, Student's *t *test.

We hypothesized that the ability of RhoB to modulate ERα expression could control the major transcriptional functions of ERα. To examine this hypothesis, we used MELN cells that express luciferase in an estrogen-dependent manner. After siB1 or siB2 transfection and E_2 _deprivation, cells were treated or not with E_2 _and luciferase activity was quantified (Figure 4A). In the absence of E_2 _(upper-right corner), the transfection of cells by both siB1 and siB2 induced a significant decrease of the luciferase activity (0.5-fold for B1 and 0.7-fold for B2). In the control cells, E_2 _treatment led to a ninefold induction of the luciferase activity. In the presence of E_2_, luciferase expression was significantly decreased for cells treated by the siB1 and siB2 sequences (respectively 3.12 and 4.07 compared with 9 AU). Nonetheless, E_2 _induction was still observed for all cells transfected by either siB1 or siB2. We extended the analysis of RhoB inhibition on two known estrogen-regulated genes, *ERα *itself described above (Figure 3D) and *PR*, using quantitative RT-PCR (Figure 4B).

Regarding *PR*, a major target gene of ERα, PR mRNA expression was induced in the presence of E_2 _(7.7-fold) in the siRNA control cells (Figure 4B). In cells treated with siB1, there was a significant decrease of PR mRNA levels as compared with their respective control, both without and with E_2 _(rates 0.5 and 0.6, respectively). Consequently, E_2 _induction was maintained for cells transfected by both siRNA control and siB1 (respectively 7.7-fold and ninefold).

Using immunocytochemistry, we confirmed that RhoB regulates ERα (Figure 3C) plus PR (Figure 4C) expression, with no dramatic changes of their subcellular localization within MCF-7 cells. PR is mainly detected in the nuclei of the untreated control. Besides, the presence of siB2 again significantly decreases IRS scores both in the absence and presence of E_2_.

Cells treated with siB2 exhibit low levels of ERα protein and mRNA in both the absence and presence of E_2 _(Figure 3B, C, D). Nonetheless, the effects of RhoB on PR expression may be supported in part by other mechanisms. We used chromatin immunoprecipitation analysis to study the effect of RhoB inhibition on the recruitment of ERα, ER transcriptional co-factors and RNA polymerase II onto *PR *gene promoter 1 hour after E_2 _stimulation (Figure 4D). Following siB1 transfection, the recruitment of RNA polymerase II to the *PR *gene promoter dramatically decreased (rate 0.3 compared with siControl). Recruitment of acetylated H3 and H4 histones were also clearly decreased by RhoB downregulation (rates 0.36 and 0.33, respectively). Moreover, RhoB downregulation induced a significant decrease in the recruitment of ER to the *PR *gene promoter (rate 0.52) paralleled by an increased in recruitment of the ERα co-repressor HDAC1 (rate 1.67). Together, these results indicate that RhoB may support to some extent ERα transcriptional activation function by interfering with its cofactor recruitment, besides the direct effect on ERα expression itself.

### RhoB induces proliferation in ER-positive but not in ER-negative breast cancer cell lines

**Figure 5:**
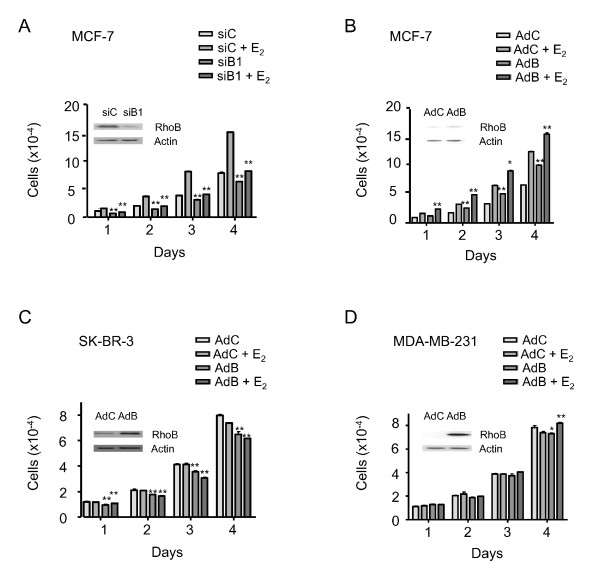
**RhoB differentially affects the proliferation of breast cancer cell lines**. Forty-eight hours after transfection or transduction, cells were seeded with estradiol (E_2_) or ethanol and counted daily. Error bars represent the mean values ± standard deviation from three independent experiments that generated triplicate data each. **(A) **MCF-7 cells were transfected with siControl (siC) or siB1. **(B) **MCF-7 cells, **(C) **SK-BR-3 cells and **(D) **MDA-MB-231 cells were transduced with adenoviral vectors (multiplicity of infection 100:1). Control western blot experiments are shown in the presence of E_2_. According to the Kruskal-Wallis test, differences were considered statistically significant at *P < 0.01 and **P < 0.001 by comparing siB1 or adenoviral vector expressing RhoB (AdB) conditions with the related control condition (either in the presence or absence of E_2_). The significance threshold was determined at 0.0125 using Bonferroni correction. AdC, adenoviral control empty vector.

The effect of RhoB on cell proliferation was evaluated in three cell lines exhibiting variable levels of expression of ERα, including MCF-7 (ERα/PR-positive), SK-BR-3 (ERα/PR-negative and p185^erbB2 ^overexpressed) and MDA-MB-231 (ERα/PR-negative and p185^erbB2^-negative). As shown in Figure 5A, B, RhoB positively regulated the proliferation of MCF-7 cells both in the absence or presence of E_2_. siRNA-mediated inhibition of RhoB expression produced a 30 to 35% decrease in MCF-7 cell proliferation as soon as 1 day after transfection, with a 40 to 46% decrease by day 4 (Figure 5A). Conversely, a significant increase in cell proliferation was observed in MCF-7 cells transduced with an adenoviral vector expressing RhoB (Figure 5B), with an increase of 15 to 28% in relative cell proliferation at day 1 that reached 22 to 49% by day 4. In contrast to these observations, under similar conditions for infection of SK-BR-3 or MDA-MB-231 cells, the adenoviral RhoB vector either slightly decreased or had no significant biological effect on cell proliferation (Figure 5C, D). The effect of RhoB downregulation was also analyzed in LCC2 cells, an E_2_-independent, tamoxifen-resistant subline of the MCF-7 cells. As for the MCF-7 cells, a significant decrease of proliferation was observed at day 4, in parallel to ERα and RhoB downregulation (see Figures S2 and S3 in Additional files 4 and 5).

In conclusion, we documented that RhoB had stimulatory effects on proliferation via ERα signaling that paralleled its effects on hormone receptor expression.

## Discussion

Although the tumor suppressor function of RhoB has been documented in many human cancers [11,12], RhoB overexpression was suggested to be associated with tumor progression in breast cancers. Using cellular and human breast tumor analytical approaches, we have shown a positive crosstalk between RhoB and ERα expression and the critical role of RhoB in regulation of the proliferation of ERα-expressing breast cancer cells.

Our data bring together three lines of evidence to support the crosstalk between ERα and RhoB. First, in human breast cancer tissues we clearly showed a strong correlation between RhoB expression and the expression of ERα and PR. Moreover RhoB expression was associated with a low tumor grade and size, suggesting that RhoB expression is correlated with good prognosis markers. The second strand of evidence came from cellular results showing that the level of RhoB controls the expression of ERα in ERα-positive breast cancer cell in the presence or absence of E_2_. The positive modulation of ERα expression by RhoB was evidenced both at the protein and mRNA levels. We showed that the ERα mRNA level is controlled by RhoB, suggesting that transcriptional regulation could play a key role in this regulation. The phosphorylation of Ser^167 ^of ERα in tumors has been related to longer overall survival whereas the phosphorylation of Ser^118 ^could be a good prognostic marker [38,39]. We found a dramatically decreased level of ERα phosphorylation at both serine sites that can be attributed to the major decrease in total ERα expression (RhoB downregulation induced no significant change in the phosphorylated/total ERα ratio). Third, we clearly demonstrated *in vivo *the RhoB control of ERα expression in MEF cells derived from a RhoB^-/- ^mouse model. This regulation is of interest in the context of the epithelial-stromal interactions, particularly given that breast adipose fibroblasts determine the expression of aromatase [40]. Further, we demonstrated that ERα controls the activation and expression of RhoB. Notably, E_2 _treatment induces an increase of active GTP-RhoB within 30 minutes, without changing RhoB expression at that time.

ERα upregulates or downregulates the transcription of hundreds of genes [41] and *PR *is a well-known ERα target gene with a major physiological role in cell proliferation. Both PR mRNA and protein expression were significantly decreased as a consequence of RhoB and related ERα downregulations. Further findings evidenced a clear decrease of the recruitment of RNA polymerase II, acetylated H3/H4 histones and ERα onto the promoter of the *PR *gene. In parallel, we described the recruitment of the major transcriptional co-repressor HDAC1, which is known to repress RhoB expression [42]. These results provide the demonstration of a regulatory role for RhoB in ERα expression and in the balance of the associated co-regulators of ERα to control transcription of its target genes. The possibility of a direct interaction of RhoB with the ERα-dependent transcriptional machinery should not be excluded since a direct interaction between ERα and RhoGDIα in breast cancer cells has been demonstrated [30] and we have shown here a strong nuclear localization of RhoB in ERα-positive tumor tissues.

In contrast to other cancer models, RhoB is critical for the proliferation of ERα-expressing breast cancer cells, suggesting its role as a positive regulator in this model. It is noteworthy that the RhoB effect is also observed on the proliferation of the ERα-positive, tamoxifen-resistant LCC2 model cell line. Inversely, in ERα-negative cell lines (SK-BR-3 and MDA-MB-231), RhoB has no effect on proliferation - thus reinforcing the idea that RhoB promotes cell proliferation through ERα expression. These results suggest that RhoB downregulation in breast cancer cells could be associated with tumor progression in parallel to ERα extinction, with a chronology that remains to be elucidated.

## Conclusion

We have demonstrated that RhoB GTPase is a key inducer of ERα and a key regulator of PR expression. RhoB acts through various complex mechanisms underlying a feed-forward loop that may control estrogen effects, including cell proliferation. Our new findings shed light on the role of RhoB in tumorigenesis involving a dual effect conferred by cellular context with a potential pro-oncogenic function in hormone-dependent breast cancer cells.

## Abbreviations

DCC: dextran-coated charcoal; DMEM: Dulbecco's modified Eagle's medium; E_2_: estradiol; ER: estrogen receptor; FBS: fetal bovine serum; IRS: ImmunoReactive Score; MEF: mouse embryonic fibroblast; PCR: polymerase chain reaction; PR: progesterone receptor; RT: reverse transcriptase; siRNA: small interfering RNA.

## Competing interests

The authors declare that they have no competing interests.

## Authors' contributions

GF and SFD-S are co-last authors of this work. CM-G and IL-M carried out the cell line experiments and reviewed the manuscript, EMa carried out western blot, luciferase and GST pull-down experiments and participated in drafting the manuscript. EMe carried out chromatin immunoprecipitation, RT-PCR, western blot and proliferation experiments and participated in drafting the manuscript. BC participated in the adenovirus experiments. YB participated in immunocytochemistry experiments. TF performed all statistical analysis. LK carried out the patient data collection. CM carried out the tissue microarray. ML-T performed the tissue microarray analysis and reviewed the manuscript. FD participated in the design of the study and reviewed the manuscript. SFD-S conceived the study, coordinated the study and drafted the manuscript. GF coordinated the study and reviewed the manuscript. All authors read and approved the final manuscript.

## Supplementary Material

Additional file 1 **Table S1 presenting clinicopathological characteristics of 113 breast cancer patients**. Click here for file

Additional file 2 **A word file presenting the supplementary materials and methods, with information for the patient population and the proliferation determination in LCC2 cells**. Click here for file

Additional file 3 **Figure S1 showing Kaplan-Meier representation of DFS for the patients according to RhoB expression (low or high) in their tumors**. Click here for file

Additional file 4 **Figure S2 showing RhoB downregulation is associated with decreases of ERα expression in three other breast cancer cell lines**. T47D, ZR75 and LCC2 cells were transfected with siControl (siC), siB1 or siB2 during 48 hours. Protein expression was then analyzed. Representative of two to three independent experiments. Click here for file

Additional file 5 **Figure S3 showing RhoB downregulation is associated with decreased proliferation in LCC2 cells**. The LCC2 cells were transfected with siControl (siC) or siB1, and the cells were seeded 48 hours after transfection and counted at day 4. Error bars represent the mean values ± standard deviation from triplicate data. Representative of two independent experiments. Click here for file
