# Supplementary material for: RhoB modifies estrogen responses in breast cancer cells by influencing expression of the estrogen receptor
Source: Breast Cancer Res. 2013 Jan 22;15(1):R6. doi: 10.1186/bcr3377 (PMC3672819; doi:10.1186/bcr3377)
Supplement: Additional file 1 — Table S1 presenting clinicopathological characteristics of 113 breast cancer patients. [file bcr3377-S1.PDF]

Medale, Supplemental Table 1

	No adjuvant tamoxifen treatment n = 51	Tamoxifen adjuvant treatment n = 62	p	Total n = 113
Age median [range]	58 [48-74]	60 [51-74]	0.1284	60 [48-74]
Lymph nodes negative<br>Lymph nodes positive<br>Missing data	25 (49%)<br>26 (51%)<br>0	14 (23%)<br>47 (77%)<br>1	0.0039	39 (34.8%)<br>73 (65.1%)<br>1
Histological size <2 cm<br>Histological size ≥ 2 cm<br>Missing data	21 (45.7%)<br>25 (54.3%)<br>5	16 (27.1%)<br>43 (72.9%)<br>3	0.0486	37 (35.2%)<br>68 (64.8%)<br>8
Grade I and II<br>Grade III<br>Missing data	31 (62%)<br>19 (38%)<br>1	34 (55.7%)<br>27 (44.3%)<br>1	0.5052	65 (58.6%)<br>46 (41.4%)<br>2
Number mitose/ mm^2 : median [range]	2.5 [0.4-13.0]	5.1 [0.4-13.0]	0.0556	3.8 [0.4-13.0]
Ductal carcinoma<br>Lobular carcinoma<br>others	44 (86.3%)<br>4 (7.8%)<br>3 (5.9%)	47 (75.8%)<br>9 (14.5%)<br>6 (9.7%)	0.4377	91 (80.5%)<br>13 (11.5%)<br>9 (8.0%)
ERα positive<br>ERα negative<br>Missing data	34 (68%)<br>16 (32%)<br>1	40 (64.5%)<br>22 (35.5%)<br>0	0.6987	74 (66.1%)<br>38 (33.9%)<br>1
PR positive<br>PR negative<br>Missing data	30(60%)<br>20 (40%)<br>1	29 (46.7%)<br>33 (53.2%)<br>0	0.1634	59 (52.7%)<br>53 (47.3%)<br>1
LVI positive<br>LVI negative<br>Missing data	11 (22.8%)<br>38 (77.6%)<br>2	12 (21.8%)<br>43 (78.2%)<br>7	0.9383	23 (221.1%)<br>81 (77.9%)<br>9
