# Supplementary material for: RhoB modifies estrogen responses in breast cancer cells by influencing expression of the estrogen receptor
Source: Breast Cancer Res. 2013 Jan 22;15(1):R6. doi: 10.1186/bcr3377 (PMC3672819; doi:10.1186/bcr3377)
Supplement: Additional file 2 — A word file presenting the supplementary materials and methods, with information for the patient population and the proliferation determination in LCC2 cells. [file bcr3377-S2.DOC]

**Materials and Methods** **of Supplemental data**

**Patient Population**

The TMA was constructed with 114 tumor tissues from a cohort of 215 patients with operable primary breast carcinoma, treated at Institut Claudius Regaud from 1980 to 1983, and enrolled in a randomized clinical trial for adjuvant tamoxifen *versus* no adjuvant treatment. This study was approved by a central ethics committee about 30 years ago and was conducted in accordance to the Declaration of Helsinki. Legal storage of any prospective trial is 15 years, consequently today there is no document available anymore to specify the name and the date of ethical approval concerning this trial. Moreover the consent was not required at the moment the study was performed. Patients received radical mastectomy, modified radical mastectomy or breast-conserving surgery. Axillary lymph nodes were routinely dissected at levels I, II and III, and lymph node metastasis was determined based on histological examination. At that time, tumors were considered as ERα positive using a biochemistry determination. For the present study, paraffin blocks of tumor tissue were available for 114 patients. Among these cases, we failed to assess immunochemistry in only 1 tumor specimen, which was therefore excluded. Histological type, Elston and Ellis tumor grade, and presence of lymphovascular invasion were newly assessed for the 113 specimens available for pathological review. Tumor size was defined as the maximum tumor diameter measured on surgical specimens. Follow-up data were available with a median of 249.9 months. Differences between treatment modality were assessed using a Chi-square or Fisher's exact test for qualitative variables and Mann-Whitney for continuous variables.

**Proliferation determination in LCC2 cells**

48h hours after siRNA transfection, cells were seeded in F-12 phenol red-free medium, containing 5% DCC-FBS and proliferation was analyzed at day 4 with the Cell Titer 96 Aqueous One solution (*Promega*), according to the manufacturer’s instructions.
