# Supplementary figures and images for: RhoB modifies estrogen responses in breast cancer cells by influencing expression of the estrogen receptor
Source: Breast Cancer Res. 2013 Jan 22;15(1):R6. doi: 10.1186/bcr3377 (PMC3672819; doi:10.1186/bcr3377)

Medale, Supplemental Figure 1

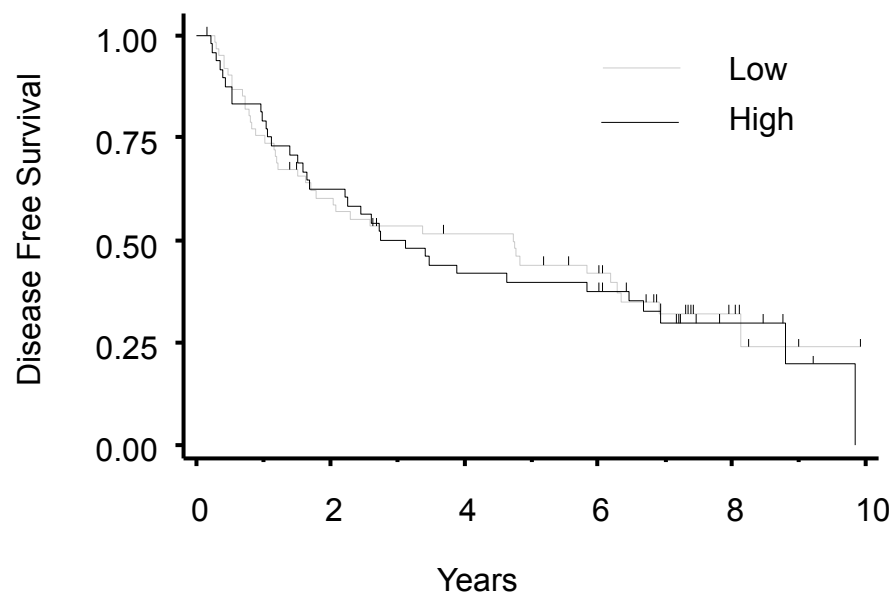

Supplement: Additional file 3 — Figure S1 showing Kaplan-Meier representation of DFS for the patients according to RhoB expression (low or high) in their tumors. [file bcr3377-S3.PDF]

Medale, Supplemental Figure 2

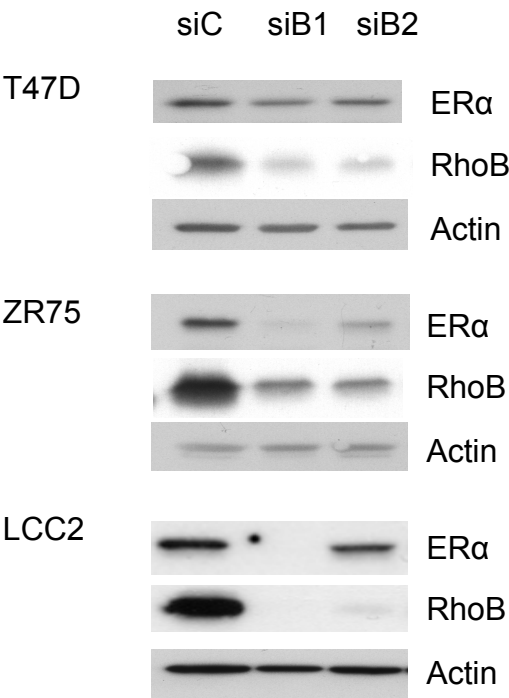

Supplement: Additional file 4 — Figure S2 showing RhoB downregulation is associated with decreases of ERα expression in three other breast cancer cell lines. T47D, ZR75 and LCC2 cells were transfected with siControl (siC), siB1 or siB2 during 48 hours. Protein expression was then analyzed. Representative of two to three independent experiments. [file bcr3377-S4.PDF]

Medale, Supplemental Figure 3

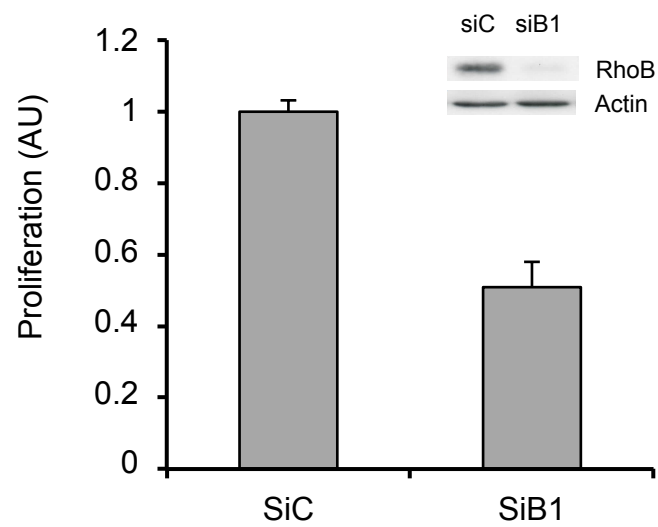

Supplement: Additional file 5 — Figure S3 showing RhoB downregulation is associated with decreased proliferation in LCC2 cells. The LCC2 cells were transfected with siControl (siC) or siB1, and the cells were seeded 48 hours after transfection and counted at day 4. Error bars represent the mean values ± standard deviation from triplicate data. Representative of two independent experiments. [file bcr3377-S5.PDF]
